# Protective Effects of Pear Extract on Skin from In Vitro and In Vivo UVA-Induced Damage

**DOI:** 10.3390/ph17050583

**Published:** 2024-05-02

**Authors:** Thomas W. Chu, Ching-Chih Ho, Yu-Jou Hsu, Yuan-Hsin Lo, Nan-Lin Wu, Yuan-Bin Cheng, Mao-Xuan Hong, Der-Chen Chang, Chi-Feng Hung

**Affiliations:** 1 Department of Dermatology, Far Eastern Memorial Hospital, New Taipei City 22060, Taiwan; tchumd@gmail.com; 2 Department of Dermatology, Eastern Virginia Medical School, Norfolk, VA 23507, USA; 3 Department of Anesthesiology, Taoyuan Armed Forces General Hospital, Longtan, Taoyuan 325, Taiwan; garyboyho@gmail.com; 4 PhD Program in Pharmaceutical Biotechnology, Fu Jen Catholic University, New Taipei City 24205, Taiwan; s16179263@gmail.com; 5 Department of Dermatology, Fu Jen Catholic University Hospital, Fu Jen Catholic University, New Taipei City 242, Taiwan; d03954@mail.fjuh.fju.edu.tw; 6 Department of Medicine, Mackay Medical College, New Taipei City 25245, Taiwan; alvin.4200@mmh.org.tw; 7 Department of Dermatology, MacKay Memorial Hospital, Taipei 10491, Taiwan; 8 Department of Marine Biotechnology and Resources, National Sun Yat-Sen University, Kaohsiung 804351, Taiwan; jmb@mail.nsysu.edu.tw (Y.-B.C.); a0933499907@gmail.com (M.-X.H.); 9 Department of Mathematics and Statistics and Department of Computer Science, Georgetown University, Washington, DC 20057, USA; chang@georgetown.edu; 10 School of Pharmacy, Kaohsiung Medical University, Kaohsiung 80708, Taiwan; 11 School of Medicine, Fu Jen Catholic University, New Taipei City 24205, Taiwan

**Keywords:** pear, skin photoaging, UVA, cytokines, skin barrier

## Abstract

The ancient Chinese medical book “Compendium of Materia Medica” records that pears can relieve symptoms of respiratory-related diseases. Previous research has shown that pear *Pyrus Pyrifolia* (Burm.f.) Nakai has antioxidant and anti-inflammatory properties. However, the anti-inflammatory, antioxidant, and anti-photoaging protective effects of *Pyrus pyrifolia* (Burm.f.) Nakai seed components have not been studied. Ultraviolet light (UV) causes skin inflammation, damages the skin barrier, and is an important cause of skin photoaging. Therefore, UV light with a wavelength of 365 nm was used to irradiate HaCaT and mice. Western blot, real-time quantitative polymerase chain reaction, and fluorescence imaging system were used to explore its anti-UVA mechanism. Dialysis membrane and nuclear magnetic resonance were used for the chemical constituent analysis of pear seed water extract (PSWE). We found that PSWE can significantly reduce UVA-induced skin cell death and mitogen-activated protein kinase phosphorylation and can inhibit the mRNA expression of UVA-induced cytokines (including IL-1β, IL-6, and TNF-α). In addition, PSWE can also reduce the generation of oxidative stress within skin cells. In vivo experimental studies found that PSWE pretreatment effectively reduced transepidermal water loss, inflammation, redness, and dryness in hairless mice. The molecular weight of the active part of pear water extract is approximately 384. Based on the above results, we first found that pear seeds can effectively inhibit oxidative stress and damage caused by UVA. It is a natural extract with antioxidant properties and anti-aging activity that protects skin cells and strengthens the skin barrier.

## 1. Introduction

Excessive sun exposure can easily cause the skin to be damaged by ultraviolet rays. It can easily lead to premature skin aging, melanin spots, wrinkles, thinning, poor skin elasticity, dilation of blood vessels, and even skin cancer. Moreover, prolonged exposure to ultraviolet rays directly inhibits the skin’s immune system, weakening the body’s resistance and causing many skin diseases to worsen [1]. A secondary mechanism for avoiding the damage of ultraviolet rays to the skin, in addition to wearing sunscreen and hats to protect the skin when going out, is to remove harmful substances related to ultraviolet damage to the skin [2]. The signal generated by cells after UV exposure is activated by the signal transduction pathway. Due to the interference of UV light, the signal pathway may lead to cell malfunction, disruption of homeostasis, changes in gene expression, regulation of cytokines, and cell cycle control loss, all of which may lead to cellular mutations and the formation of cancer cells [3,4].

Mitogen-activated protein kinases (MAP kinases) is a serine/threonine kinase activated through the dual phosphorylation of threonine and tyrosine residues that regulates gene expression, mitosis, motility, metabolism, and death. Three family members of protein kinases include p38 kinases, extracellular regulated kinases (ERK1/2), and c-Jun N-terminal kinases (JNK) [5]. ERK could be activated in response to growth stimulation and treatment with phorbol esters. JNK is also activated in response to growth factor signaling but is also activated in response to cellular stress (such as UV and inflammatory cytokines) as in p38 [6,7]. Activation of both JNK and p38 also plays a critical role in UVA-mediated activator protein 1 (AP-1) transactivation and c-Fos expression in these human keratinocyte cells [8]. Additionally, accumulating evidence indicated that UV-induced ROS could promote the production of pro-inflammatory cytokines such as tumor necrosis factor-α (TNF-α), which were directly related to the mitogen-activated protein kinases and the nuclear factor-κB (NF-κB) signaling pathways [9,10]. Both transcription factors NF-κB and AP-1 are known to regulate various genes encoding inflammatory mediators [11]. Therefore, mitogen-activated protein kinases and the NF-κB signaling pathways have a very important regulatory role in many cellular responses induced by UV light.

Pears are one of the oldest plants cultivated by humans [12]. According to the ancient Chinese medicine book “Compendium of Materia Medica”, pears can be used to relieve symptoms of respiratory-related diseases [13]. *Pyrus pyrifolia* (Burm. f) Nakai is a plant belonging to the Rosaceae family. It is a perennial deciduous fruit tree with many names, such as Asian pear, Korean pear, and Japanese pear. Previous studies have shown that the fruit and skin of *Pyrus pyrifolia* (Burm.f) Nakai can be used for anti-diabetic, hypolipidemic, anti-inflammatory, and anti-cancer effects, cough relief, chronic skin diseases, asthma, heart protection, and skin lightening [14]. Arbutin and chlorogenic acid are the main phenolic components in Oriental pear [14], and the concentration of chlorogenic acid in the core is greater than that in the peel [15]. It is also known that the mechanism of action of the leaf extract of *Pyrus ussuriensis* Maxim applied in atopic dermatitis (AD) can effectively down-regulate the pro-inflammatory hormones TNF-α, IL-6, and IL-1β produced after the stimulation of HaCaT cells [16]. Extracts from different parts of the pear have a potential clinical application value due to their biological activities against various diseases [12,13]. Arbutin has a whitening effect because it can inhibit the activity of tyrosinase and thereby reduce the formation of melanin. Pears are a naturally rich source of arbutin. Therefore, pears also have the effect of whitening skin [14]. However, the mechanisms of anti-UV and anti-aging skin remain unclear. Therefore, this study mainly explored the pharmacological mechanism of anti-UVA of pear seed water extract (PSWE) and hoped to be widely used in anti-skin photoaging in the future.

## 2. Results

### 2.1. NMR Analysis of Pear Water Extract

**Figure 1:**
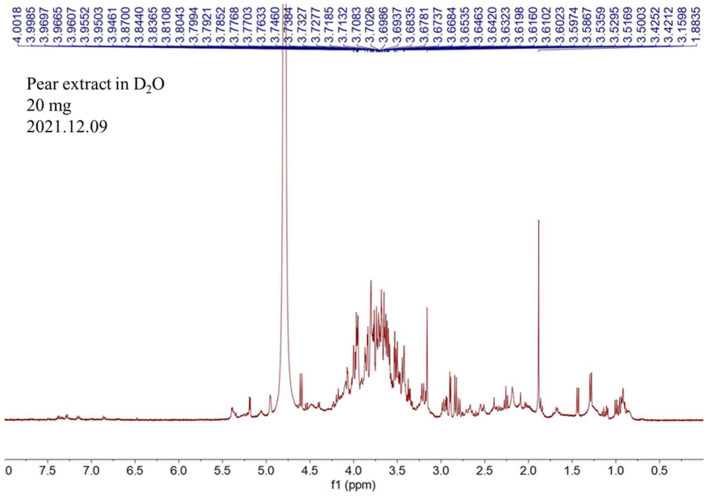
^1^H NMR spectrum of the water extract of pear.

In the ^1^H NMR spectrum (Figure 1), peaks are mainly found around 3–4 ppm, which indicate that the water extract of pear seed contains a lot of oxygenated methines (anomeric proton signals). This information suggested that the main components of the water extract of pear are polysaccharides.

### 2.2. Determination of Molecular Weight of Pear Water Extract

**Figure 2:**
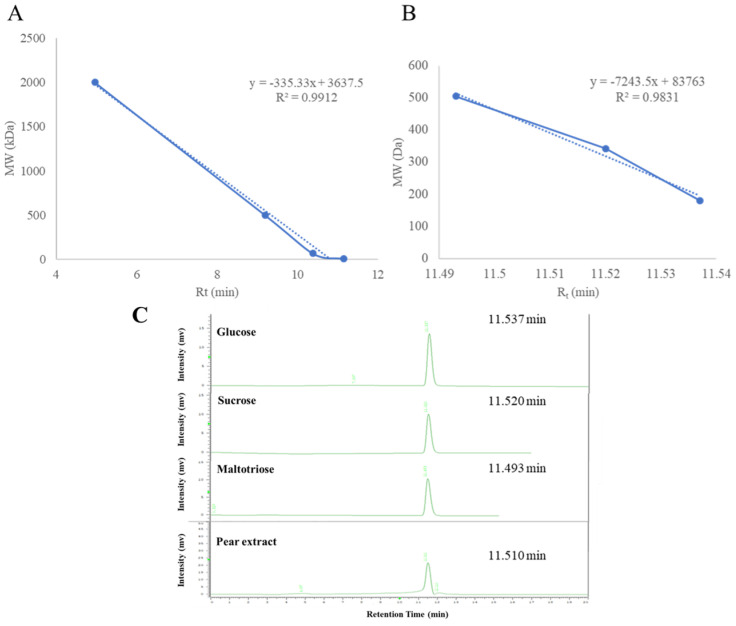
(**A**) Calibration curves of standards of different molecular weights of 2000, 500, 70, and 10 kDa. (**B**) Calibration curves of standards of glucose, sucrose, and maltotriose. (**C**) The retention time of glucose, sucrose, maltotriose, and the active portion of pear water extract in size-exclusion HPLC column.

The water extract of pear seed was dialyzed by a dialysis membrane, and two portions with different molecular weights were obtained. The UVA protective effects of the inner (MW > 10 kD) and outer (MW < 8 kD) portions were evaluated. The outer portion showed better activity. Thus, this portion was chosen for further analytical experiments to determine the ingredients of polysaccharides. Dextrans of different molecular weights (2000, 500, 70, and 10 kDa), glucose, sucrose, and maltotriose were purchased and injected into a size-exclusion HPLC column, and the results are shown in Figure 2. The active portion of PSWE was detected at the retention time of 11.5 min, which suggested that the molecular weight of PSWE is about 384.6.

### 2.3. Cytotoxicity and Protective Effects of PSWE against UVA on HaCaT Cells

**Figure 3:**
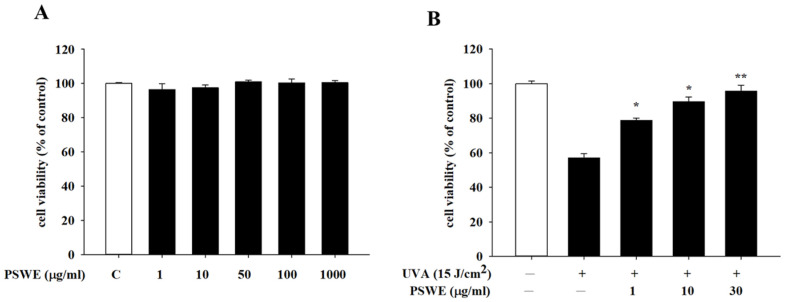
The effects of HaCaT cells treated with different concentrations of PSWE on cell viability (**A**) and UVA protection (**B**). Cell viability of HaCaT cells after pretreatment with different concentrations of PSWE (1, 10, 50, 100, and 1000 μg/mL). HaCaT cells were treated with different concentrations of PSWE at 37 °C for 24 h. Values represent the mean ± SEM from at least three experiments. * *p* < 0.05 and ** *p* < 0.01 compared with group that was only treated with UVA and not pretreated with extract.

MTT was used to detect the effect of PSWE on the survival rate of human keratinocyte cell line (HaCaT) against ultraviolet A damage. First, HaCaT cells were pretreated with different concentrations of PSWE (1–1000 µg/mL), and the cell viability and cytotoxicity of HaCaT were detected 24 h later. The results showed that PSWE treatment groups with different concentrations did not cause cell death at any concentration (Figure 3A). For further pharmacological mechanism experiments, we selected 1 µg/mL, 10 µg/mL, and 30 µg/mL of PSWE as follow-up concentrations required for the experiments. Furthermore, we wanted to know whether PSWE has a protective effect against photoaging after cells are stimulated by UVA radiation. Results showed that the cell viability of HaCaT cells was reduced by approximately 40% after being irradiated by UVA (15 J/cm^2^). It was further found that when HaCaT were pretreated with PSWE and then irradiated with UVA, the cell viability was higher than that of the untreated PSWE group, and this protective effect increased with the increase in PSWE concentration (Figure 3B).

### 2.4. PSWE Inhibits the Phosphorylation of UVA-Induced MAP Kinase and NF-κB Pathway in HaCaT

**Figure 4:**
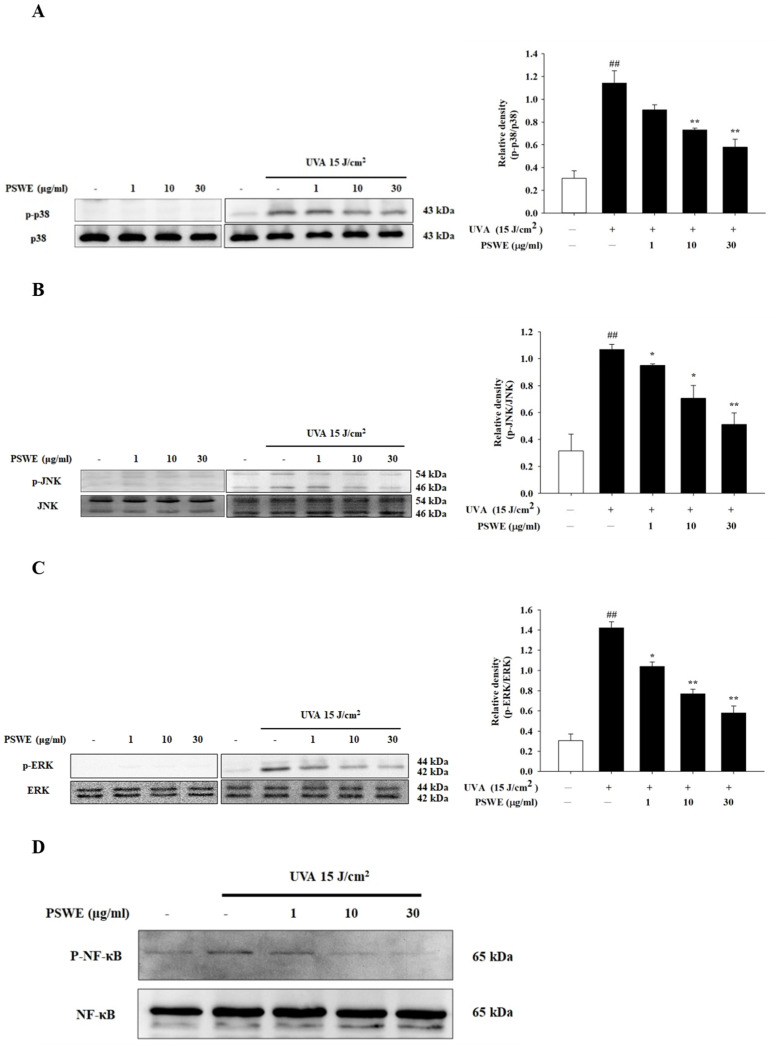
PSWE reduced UVA-induced phosphorylations of p38 (**A**), JNK (**B**), ERK (**C**), and NF-κB (**D**) in HaCaT cells. HaCaT cells were pretreated with different concentrations of PSWE (1, 10, and 30 μg/mL) for 1 h, and then, the cells were irradiated with UVA (15 J/cm^2^). Western blots were analyzed quantitatively (right panel). Values represent the mean ± SEM from three independent experiments. ## *p* < 0.01 compared with the group that was not treated with UVA and extract; * *p* < 0.05 and ** *p* < 0.01 compared with group that was only treated with UVA and not pretreated with extract.

UVA irradiation on HaCaT can cause various pathological phenomena due to immune and inflammatory reactions. Therefore, we investigated whether PSWE could inhibit these responses and pathophysiological phenomena. Among them, the MAP kinase signaling pathway is closely related to the UVA activation pathway, which can regulate various immune and inflammatory responses of the skin cell signaling pathway. In this experiment, HaCaT cells were pretreated with different concentrations of PSWE (1 µg/mL, 10 µg/mL, 30 µg/mL) for 1 h, and then irradiated with UVA (15 J/cm^2^). Analysis of the protein phosphorylation of p38, ERK, and JNK in this pathway showed that the HaCaT group treated with different concentrations of PSWE alone did not affect the phosphorylation of p38, ERK, and JNK proteins (Figure 4A–C). However, p38, ERK, and JNK could be found in the UVA treatment group. The phosphorylation of JNK protein was significantly increased. In the PSWE pretreatment group, it was observed that the protein phosphorylation of p38, ERK, and JNK decreased with the increase in PSWE concentration (Figure 4A–C). NF-κB is a protein complex that controls DNA transcription, and its dysregulation has been linked to various diseases including cancer, inflammation, and aging. NF-κB can be activated by ROS released after UV irradiation. Activated NF-κB translocates to the nucleus and induces the expression of pro-inflammatory cytokines. Therefore, we investigated whether it could also effectively inhibit the expression of NF-κB. UVA stimulation indeed induces the significant phosphorylation of NF-κB. PSWE pretreatment showed that, as the concentration increased, the phosphorylation reaction gradually decreased (Figure 4D).

### 2.5. PSWE Down-Regulated the mRNA Expressions of Pro-Inflammatory Hormones IL-1β, IL-6, and TNF-α Produced by HaCaT Cells Stimulated by UVA

**Figure 5:**
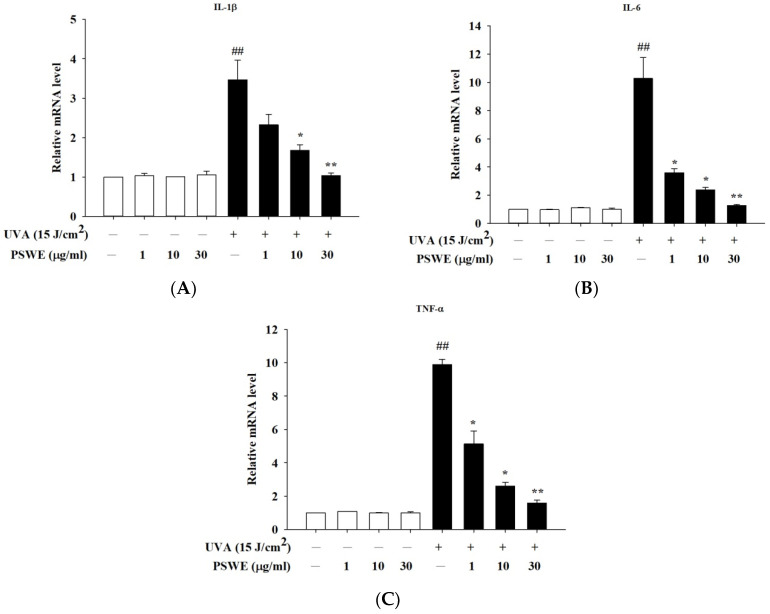
PSWE inhibited UVA-induced mRNA expression levels of IL-1β, IL-6, and TNF-α in HaCaT cells. The effect of PSWE on the mRNA expression levels of cytokines in UVA-stimulated HaCaT cells. HaCaT cells were pretreated with different concentrations of PSWE (1, 10, and 30 μg/mL) for 1 h, and then, the cells were treated with UVA (15 J/cm^2^) for 24 h. Total RNA was isolated, and the mRNA expression levels of (**A**) IL-1β, (**B**) IL-6, and (**C**) TNF-α were determined using qPCR. Values represent the mean ± SEM from three independent experiments. ## *p* < 0.01 compared with the group that was not treated with UVA and the extract; * *p* < 0.05 and ** *p* < 0.01 compared with group that was only treated with UVA and not pretreated with the extract.

Skin aging and inflammation caused by UVA mainly accelerate the severity of the disease through the trigger hormones produced by skin epidermal cells. Previous studies have shown that UVA acts on skin cells to induce the production of IL-1β, IL-6, and TNF-α, causing skin-related inflammatory reactions. Therefore, in this study, we used PSWE to pretreat HaCaT cells, and then UVA After 15 J/cm^2^ stimulation, RT-qPCR was used to detect whether PSWE could change the expression of IL-1β, IL-6, and TNF-α. From the experimental results, it was evident that, in the group treated with PSWE alone, the observed mRNA expression of cytokines was not affected. The mRNA expression of UVA that induces inflammatory response was significantly increased. In groups pretreated with different concentrations of PSWE (1 µg/mL, 10 µg/mL, 30 µg/mL), it was observed that the inhibitory effects of IL-1β, IL-6, and TNF-α mRNA expressions were more significant with the increase in PSWE concentration (Figure 5).

### 2.6. PSWE Inhibits the Effect of UVA-Induced ROS Production in HaCaT Cells

**Figure 6:**
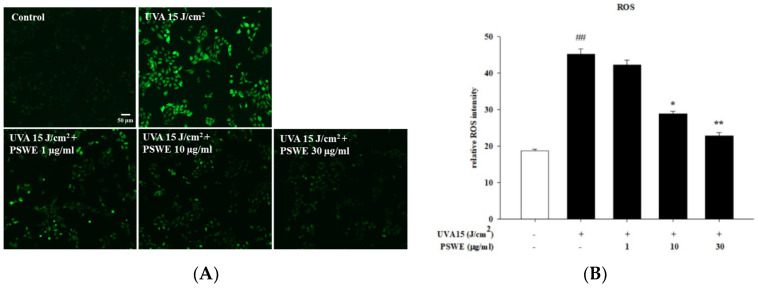
Effect of PSWE inhibited UVA-induced ROS generation in human keratinocytes cells. Effect of PSWE on UVA-induced ROS generation in HaCaT cells. HaCaT cells were pretreated or not with PSWE (10 and 30 μg/mL) for 1 h, and cells were stained with DCFH-DA for 30 min then irradiated with UVA (15 J/cm^2^). After incubation for 15 min, the cells were washed with PBS. (**A**) The intracellular levels of ROS were imaged on a fluorescence microscope. (**B**) Quantitative analysis of fluorescence. Values represent the mean ± SEM from three independent experiments. ## *p* < 0.01 compared with the group that was not treated with UVA and the extract; * *p* < 0.05 and ** *p* < 0.01 compared with group that was only treated with UVA and not pretreated with the extract.

UVA exposure is primarily associated with oxidation and inflammation. ROS plays a very important role. To discuss the mechanism of oxidative stress, cells were stained with DCFH-DA using a fluorescence microscope to detect ROS production in cells. It can be seen from the experimental results that, when the concentration of PSWE is 1 μg/mL, the production of ROS caused by UVA is slightly reduced. As the concentration of PSWE increases to 10 µg/mL and 30 µg/mL, the production of inhibited ROS also increases significantly. It can be observed in Figure 6 that the group treated with UVA 15 J/cm^2^ alone has a significant increase in the production of ROS in HaCaT cells. Compared with the group treated with PSWE, it can be proved that PSWE can effectively prevent the production of ROS after UVA irradiation.

### 2.7. The Protective Effect of PSWE on Skin Damage Caused by UVA

**Figure 7:**
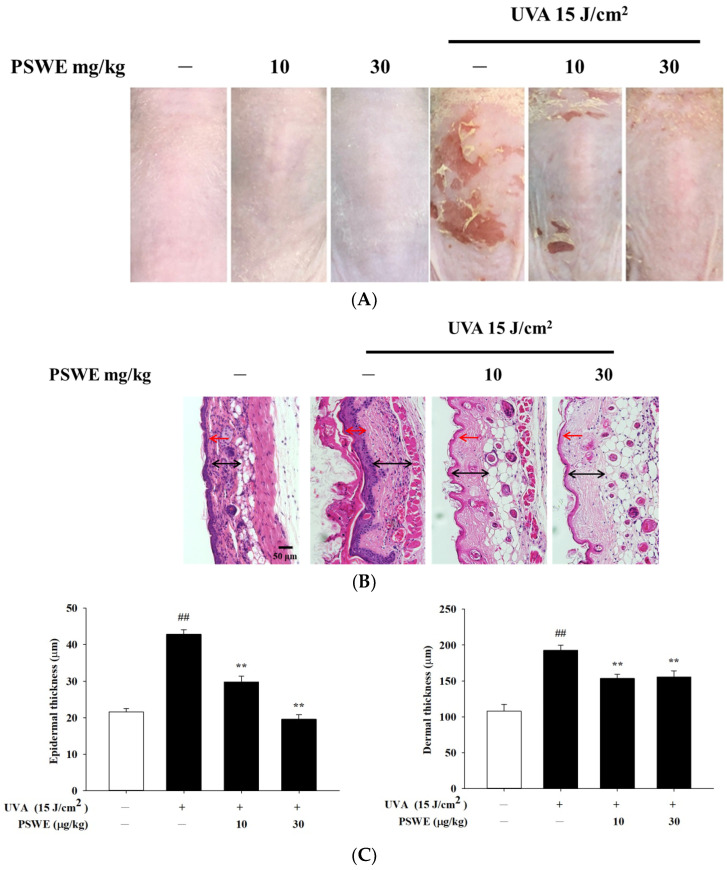
(**A**) PSWE reduces the dryness and breakage of the dorsal skin of mice caused by UVA—phenotypic presentation of mouse skin after 15 days of treatment. (**B**) Histopathological variation due to UVA induction was evaluated using hematoxylin–eosin staining. Red arrow: epidermis. Black arrow: dermis (scale bar, 50 μm). (**C**) Quantitative analysis of epidermal thickness and dermis thickness. Values represent the mean ± SEM from at least five independent experiments. ## *p* < 0.01 compared with the group that was not treated with UVA and the extract; ** *p* < 0.01 compared with group that was only treated with UVA and not pretreated with the extract.

The changes in the skin appearance of the mice were recorded by taking photos. Firstly, the mice were divided into 6 groups, namely, the control group, the PSWE control groups with concentrations of 10 mg/kg and 30 mg/kg, the irradiation by UVA 15 J/cm^2^ group, and UVA irradiation after pretreatments of 10 or 30 mg/kg PSWE groups. The experimental results showed that feeding different concentrations of PSWE alone did not cause irritation and damage to the mice. The skin appearance was not obvious compared with the control group. However, in the group irradiated with UVA alone, the dorsal skin of the mice began to have peeling and inflammation after the 3rd day. As the number of days of the experiment increased, the phenomenon became more serious. After using PSWE tube feeding, the desquamation and inflammation levels were significantly improved (Figure 7A). H&E staining images of tissue showed that the experimental group irradiated with UVA 15 J/cm^2^ alone had significantly thickened epidermis and dermis compared with the control group. If treated with different concentrations of PSWE, it can be observed that PSWE can slow down the thickening of the epidermis and dermis, and effectively improve inflammation, redness, and peeling (Figure 7B,C).

### 2.8. PSWE Improved UVA-Induced Skin Inflammation in Hairless Mice

**Figure 8:**
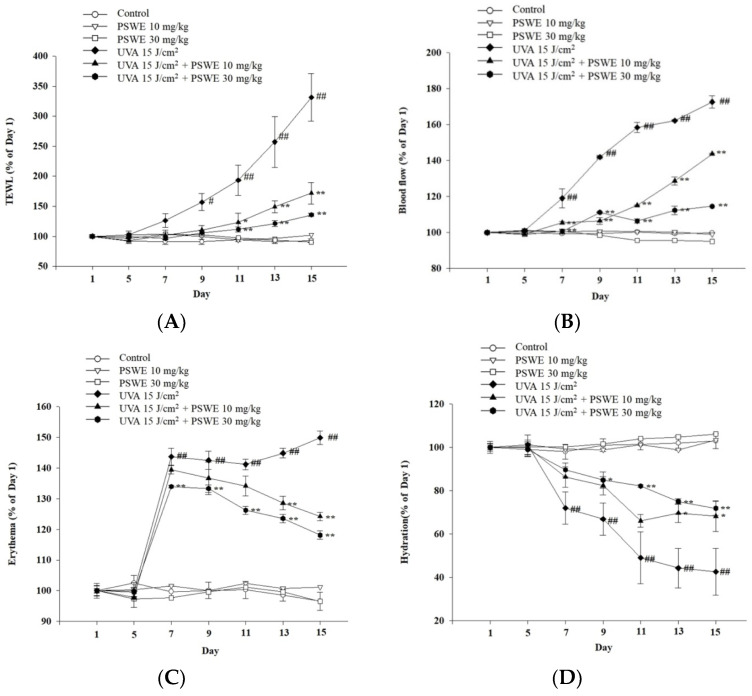
Change in physiological parameters of UVA-induced BALB/cAnN.Cg-Foxn1nu/CrlNarl mouse skin after treatment with PSWE. Analysis of the effect of the change in (**A**) transepidermal water loss (TEWL), (**B**) blood flow, (**C**) erythema, and (**D**) hydration. Values represent the mean ± SEM from at least three independent experiments. # *p* < 0.05; ## *p* < 0.01 compared with the group that was not treated with UVA and the extract; * *p* < 0.05 and ** *p* < 0.01 compared with group that was only treated with UVA and not pretreated with the extract.

After exposure to ultraviolet rays, the barrier function of the skin is damaged. Therefore, in addition to taking photos to record the protective effect of PSWE on ultraviolet rays, this experiment uses a multi-functional skin detector to detect changes in various physiological parameters of the skin surface, including transepidermal water loss (TEWL), blood flow, erythema, and hydration of skin (Figure 8). The results showed that without UVA irradiation, PSWE had no change in the above physiological values compared with untreated PSWE (Figure 8). After the third day of ultraviolet A irradiation, TEWL increased significantly, showing that the skin tissue was damaged. However, TEWL is significantly reduced after pretreatment with PSWE. Results show that PSWE can slow down UVA-induced transepidermal water loss (Figure 8A). It has the same effect on blood flow. It can be seen that PSWE can also slow down skin inflammation after UVA irradiation (Figure 8B). The results also showed that, in the PSWE pretreatment group, the effect of UV on skin erythema was reduced. This shows that PSWE can indeed effectively reduce UVA-induced erythema and inflammation (Figure 8C). Additionally, the results also showed that UVA has a significant impact on reducing skin hydration, leading to dry skin. The results also found that, after pretreatment with PSWE, the skin’s moisturizing ability slowly recovers (Figure 8D).

### 2.9. PSWE Reduces the Expression of mRNA That Promotes Inflammatory Cytokines IL-1β, IL-6, and TNF-α Induced by UVA in Hairless Mice

**Figure 9:**
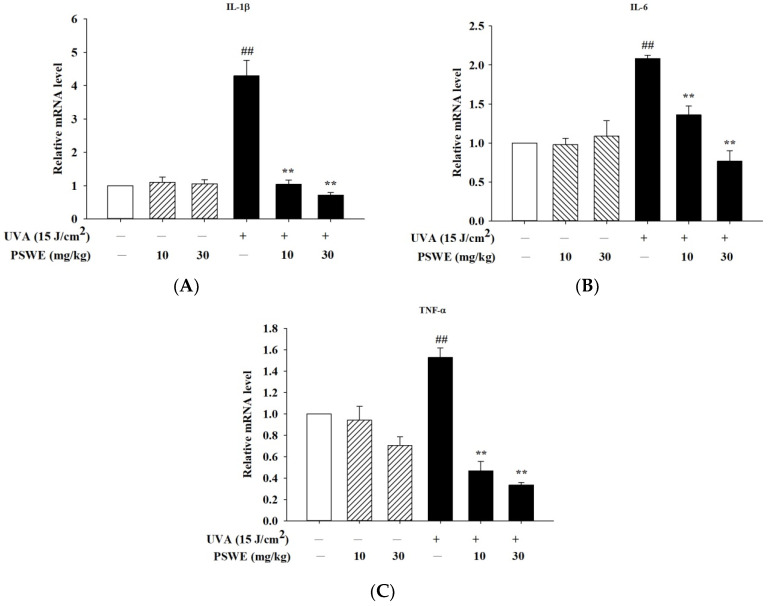
PSWE inhibited UVA-induced cytokine of mRNA expression of IL-1β, IL-6, and TNF-α in the skin tissue of BALB/cAnN.Cg-Foxn1nu/CrlNarl mice. The effect of PSWE on the mRNA expression levels of cytokines in UVA-stimulated mice. Mice were pretreated with different concentrations of PSWE (10 and 30 mg/kg) for 15 days, and then, the mice were irradiated with UVA (15 J/cm^2^) five times. Total RNA was isolated, and the mRNA expression levels of (**A**) IL-1β, (**B**) IL-6, and (**C**) TNF-α were determined using qPCR. Values represent the mean ± SEM from three independent experiments. ## *p* < 0.01 compared with the group that was not treated with UVA and the extract; ** *p* < 0.01 compared with group that was only treated UVA and not pretreated with the extract.

Skin aging and inflammation caused by UVA mainly accelerate the severity of the disease through pro-inflammatory cytokines produced by skin epidermal cells. In vitro studies have found that UVA induces the production of IL-1β, IL-6, and TNF-α, causing skin-related inflammatory reactions in keratinocytes. Therefore, we pretreated hairless mice with oral PSWE before UVA irradiation in vivo studies. The results have shown that the mRNA expression of cytokines was not affected in the group treated with PSWE alone, and the mRNA expression was increased after UVA irradiation. After oral treatments of PSWE (10 mg/kg and 30 mg/kg), it was observed that the mRNA expressions of IL-1β, IL-6, and TNF-α all had more significant inhibitory effects as the concentration increased (Figure 9).

### 2.10. PSWE Down-Regulates Phosphorylation of the MAP Kinase Pathway in Hairless Mice Induced by Ultraviolet A

**Figure 10:**
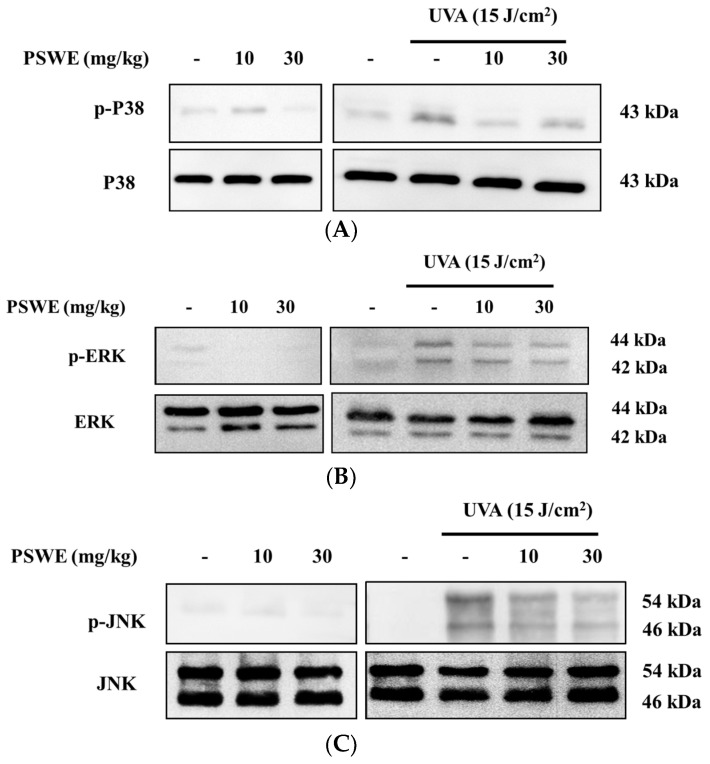
PSWE inhibited UVA-induced MAP kinase phosphorylation (**A**) P38, (**B**) ERK, and (**C**) JNK in BALB/cAnN.Cg-Foxn1nu/CrlNarl mice.

Previous studies have shown that, in the immune and inflammatory responses, the MAP kinase signal transduction pathway is activated [17]. Therefore, we wanted to understand the effect of PSWE on the activation of the MAP kinase pathway in mice after UVA irradiation. Because in vitro studies have confirmed that PSWE can effectively regulate the phosphorylation of the three main proteins, we want to confirm this result through in vivo experiments. The results show that the phosphorylation of p38, ERK, and JNK proteins is unaffected in mice treated with different concentrations of PSWE. In contrast, the group irradiated with UVA alone exhibited an increased phosphorylation of MAP kinases. In the groups treated with different concentrations of PSWE, it can be observed that the protein phosphorylation of p38, ERK, and JNK decreases as the concentration of PSWE increases (Figure 10).

## 3. Discussion

**Figure 11:**
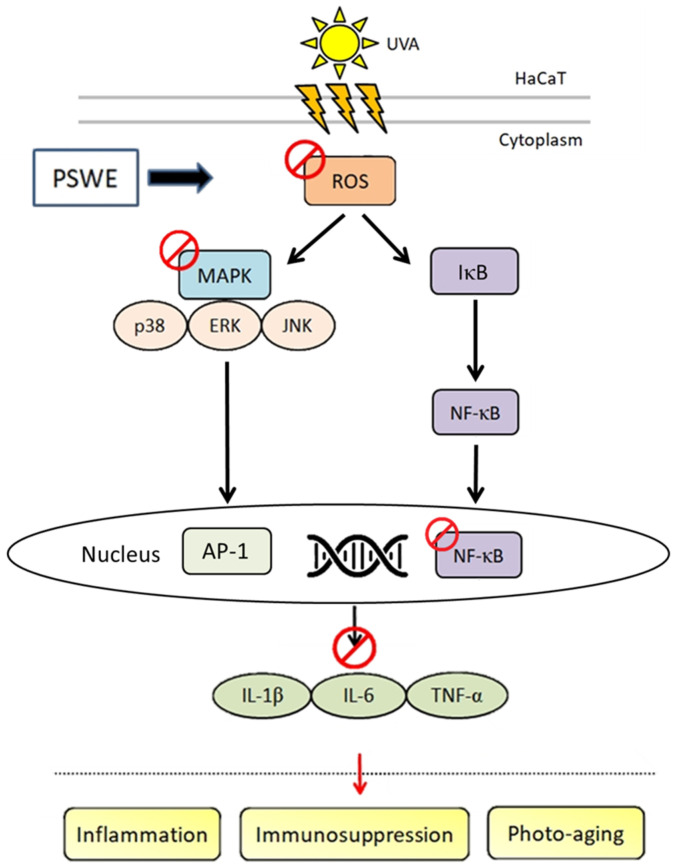
Mechanisms by which PSWE attenuates UVA-induced photoaging in HaCaT cells. UVA causes ROS production, activates MAP kinase, further causes NF-κB to translocate to the nucleus, and produces a variety of cytokines including IL-1β, IL-6, and TNF-α. The production of cytokines can lead to skin inflammation, immune effects, and even photoaging. Results show that PSWE inhibits the production of ROS, MAP kinase, and the aforementioned cytokines, which can prove that PSWE has anti-photoaging effects.

Ultraviolet rays (UV) are invisible light rays that exist in nature. They are mainly divided into three parts according to their wavelength, UVA (320–400 nm), UVB (290–320 nm), and UVC (100–280 nm). About 95% of ultraviolet rays are UVA, which has low energy and strong penetrating power. It can penetrate glass, clothes, and even penetrate the epidermis of the skin to penetrate deep into the dermis of the skin, destroying collagen fibers and elastic fibers [18]. UVA can cause skin to tan and produce many free radicals, thereby promoting accelerated skin aging. For those who are often exposed to UVA, their skin will become loose and wrinkled, causing chronic and lasting damage to the skin [19,20]. UVB will thicken and turn skin cuticles red and increase the risk of skin cancer [21,22]. Although the energy of UVB is stronger than that of UVA, it is easier to protect oneself from it. UVC is mostly isolated by the ozone layer in the atmosphere. Only a very small amount reaches the ground and generally has little effect on the human body. However, the ozone layer has been continuously damaged in recent years, and the possibility of UVC harming the human body has gradually increased [23]. It has been proved that the antioxidant, anti-inflammatory, and anti-cancer biological activities of different varieties of pears are mainly due to polyphenols, and the antioxidant capacity of various parts is also different [24]. Among them, chlorogenic acid and arbutin are the main components of polyphenols in pears [15,25]. Therefore, in this study, we utilized the bioactivity of pear seed extract (PSWE) to further explore the protective effects of damage caused by UVA and oxidative stress (Figure 11).

It can be seen from the research results that PSWE can effectively inhibit UVA damage to human keratinocyte cell line at low concentrations, and the higher the PSWE concentration, the stronger the protective effect against UVA-induced damage and cell death. Past studies have shown that the damage to skin cells after UVA irradiation is mainly carried out through reactive oxygen species, and will induce the activation of downstream signal transmission pathways, resulting in inflammation and destruction of skin tissue [26,27]. The results of this study show that PSWE can effectively inhibit the phosphorylation of p38, ERK, and JNK in the MAPK pathway caused by UVA-stimulated ROS production. Inhibition increases with increasing PSWE concentration. Subsequently, we also explored the antioxidant effect of PSWE on the generation of ROS. The results showed that the amount of ROS generated increased significantly after UVA irradiation. As the concentration of PSWE added increases, the amount of ROS generated is effectively suppressed and significantly reduced. Numerous studies in the past have shown that antioxidant and anti-inflammatory compounds can be found in fruits and vegetables, including phenolics, carotenoids, and anthocyanins [28,29]. Bioactive compounds such as flavonoids, tannins, silymarin, catechins, gallic acid, chlorogenic acid, and arbutin can prevent damage caused by free radicals [30,31,32,33,34]. Antioxidants can delay or inhibit oxidative damage caused by reactive oxygen species. Many phenolics use this effect to play a significant role in delaying aging, reducing inflammation, and preventing certain cancers [34,35]. In previous studies, almost all the bioactive compounds in the skin of pear were phenolic compounds [36]. In recent years, there has been related literature on the photoprotective effect of polysaccharides including reed rhizome polysaccharides, ganoderma polysaccharides, astragalus polysaccharides, and wolfberry crude polysaccharides on UV-induced skin cell damage [37,38]. Therefore, this study used Varian MR-400 FT-NMR to analyze the components of PSWE and found that the biological activity was mainly polysaccharides. Therefore, it may be that the antioxidant and anti-inflammatory protective mechanisms of PSWE do not use polyphenols as the main biologically active compounds in the previous literature. Whether polysaccharides are the compounds that mainly promote antioxidant and anti-inflammatory effects needs to be clarified in further research.

UV rays are the most serious factor in skin aging. They directly destroy the skin’s collagen. Whether it is winter or summer, ultraviolet rays must be well isolated to slow down the destruction of skin collagen. Therefore, sun protection is the most basic and important basis for protecting the skin. When choosing sunscreen products, the PA coefficient is related to the UVA-blocking effect [39]. The principle of sun protection is divided into two categories. The protective mechanism of physical sunscreen is to reflect, scatter, and refract ultraviolet rays to protect the skin from damage. The chemical protection mechanism is to absorb and convert UV rays into heat energy to reduce skin damage. Although chemical sunscreen products have a very good protective effect on UVA, the chemical ingredients in them can easily penetrate the skin and cause allergic irritation, which is not safe. And some ingredients can also damage the natural environment. Therefore, it is necessary to seek safer sunscreen ingredients and protect the natural environment. Studies have shown that natural antioxidant topical formulations with a sun protection factor (SPF) including lignin, melanin, silymarin, etc. have been added to natural sunscreens [40]. Through the in vivo test of natural pear seed extract, the protective effect of PSWE on UVA can be quickly and effectively proved by PSWE feeding. There is an opportunity to apply natural pear seed extract in sunscreen products in the future.

In the in vivo study, photos and records of the dorsal skin of mice showed that the dorsal skin of mice treated with PSWE alone did not cause any irritation, and the pretreatment of PSWE could effectively slow down dryness and peeling after UVA irradiation. The measurement of skin TEWL can be used as a common indicator of the barrier function of the stratum corneum of the skin and can detect whether the protective function of the skin is damaged in time [41]. When the mouse was exposed to UVA, it can be seen from the results that, the higher the TEWL value, the greater the water loss from the stratum corneum. After the pretreated PSWE mice were irradiated with UVA, the TEWL value slowed down. Through the values of erythema and blood flow, we know that PSWE can effectively slow down the redness and inflammation caused by the UVA stimulation of the skin. In addition, it can be observed through the hydration value that PSWE can protect the skin from dryness caused by UVA exposure. Therefore, the results of in vivo experiments are sufficient to confirm the validity of in vitro experiments.

From the above results, it can be known that the natural product PSWE helps inhibit the oxidation and inflammatory reactions caused by UVA irradiation. It can be used as an effective natural ingredient for preventing photoaging in the future.

## 4. Materials and Methods

### 4.1. Analysis of the Chemical Content of Pear Water Extract

Pear seeds are obtained from ripe pears and dried thoroughly before extraction. The seeds are then ground into a fine powder. The appropriate amount of deionized water was added according to the required proportion, then stirred and shaken thoroughly. After soaking and centrifugation, the mixture was filtered to separate the liquid extract and the solid residue. The extract was concentrated using a freeze-dryer. The concentrated extracts protect from light and have increased stability and activity.

The nuclear magnetic resonance (NMR) spectrum was acquired by using a Varian (Palo Alto, CA, USA) MR-400 FT-NMR spectrometer at room temperature. Samples were measured by deuterium oxide. The HPLC analyses were achieved using a Hitachi (Tokyo, Japan) 5110 pump with 5430 diode array detector equipped with a Thermo Scientific (Waltham, MA, USA) biobasic SEC-1000 column (300 × 7.8 mm, 5 μm). Dialysis of pear water extract was performed by a Repligen (Waltham, MA, USA) Spectra/Por dialysis membrane (MWCO: 8–10 kD). Amersham Pharmacia Biotech (Uppsala, Sweden) dextrans T-10, T-70, and T-500, Thermo Scientific maltotriose (MW = 504.4), Showa (Tokyo, Japan) sucrose (MW = 342.3), and Merck (Darmstadt, Germany) glucose (MW = 180.2) were used as standards for molecular weight determination.

### 4.2. Cell Line Culture

The human epidermal keratinocyte line (HaCaT) was used for in vitro experiments. The HaCaT cell line was provided by Dr. Nan-Lin Wu, Department of Dermatology, Mackay Hospital. HaCaT cells were cultured in a 75T flask containing 10% fetal bovine serum (FBS) and Dulbecco’s Modified Eagle Medium (DMEM) with 1% antibiotics. The cells were placed in an incubator at 37 °C and 5% CO_2_, and the cells were allowed to grow to about 80–90% saturation, and then, the cells were subcultured.

### 4.3. Ultraviolet A (UVA) Irradiation

A total of 5 × 10^4^ HaCaT cells were evenly mixed in DMEM medium containing 10% fetal bovine serum and 1% antibiotics and cultured in a 24-well plate, and placed in a 37 °C, 5% CO_2_ incubator for 24 h. Then, they were cultured with PBS and starved for a day. After the cells were pretreated with different concentrations of PSWE for 1 h, they were washed once with PBS. The cells were irradiated with UVA in 1.5 mL of PBS, and it took about 1 h for the energy to accumulate to 15 J/cm^2^. The UV irradiation system used was a Bio-Sun system illuminator (Vilber Lourmat, France). The emission peak is 365 nm (UVA), and the irradiation distance is 10 cm. The cells were then cultured in the incubator and taken out at different times for different experiments.

### 4.4. 3-(4,5-Dimethylthiazol-2-yl)-2,5-diphenyl-2H-tetrazolium (MTT) Assay

A total of 5 × 10^4^ HaCaT cells were evenly mixed in DMEM medium. A 500 µL volume was added to 24-well plates in sequence and placed in an incubator at 37 °C with 5% CO_2_ for 24 h, until the cells were attached to the culture plate before starting the experiment. Firstly, the cells were treated with different concentrations of drugs for 24 h, and then, MTT with a ratio of 1:9 was added to the cells without fetal bovine serum (add 300 µL to each well). After 24 h of reaction, DMSO was added to dissolve the blue-purple crystals, and the product had a maximum absorption at 550 nm. An ELISA reader was used to measure the survival rate of cells after drug treatment.

### 4.5. 2′,7′-Dichlorofluorescein Diacetate (DCFH-DA) Assay

When DCFH-DA enters cells, DCFH-DA is easily hydrolyzed by intracellular esterase to produce DCFH. Due to the presence of reactive oxygen species (ROS), DCFH is converted into the highly fluorescent compound 2′,7′ dichlorofluorescein (DCF). Therefore, as long as the fluorescence intensity is measured, the content of ROS in the cytoplasm can be known [42]. HaCaT cells are cultured at 5 × 10^4^ cells per well for 24 h, and then, the cells are pretreated with different concentrations of PSWE for 1 h. DCFH-DA was added to the cells and incubated at 37 °C for 30 min. Then, cells were washed once with PBS, and then, 1.5 mL of PBS was added. Cells were irradiated by UVA (total energy: 15 J/cm^2^), and then, the cells were placed in an incubator for 15 min. The fluorescence images were observed under a fluorescence microscope, and the fluorescence intensity in the images was analyzed using Image J 1.54i.

### 4.6. In Vivo Experiments

**Figure 12:**
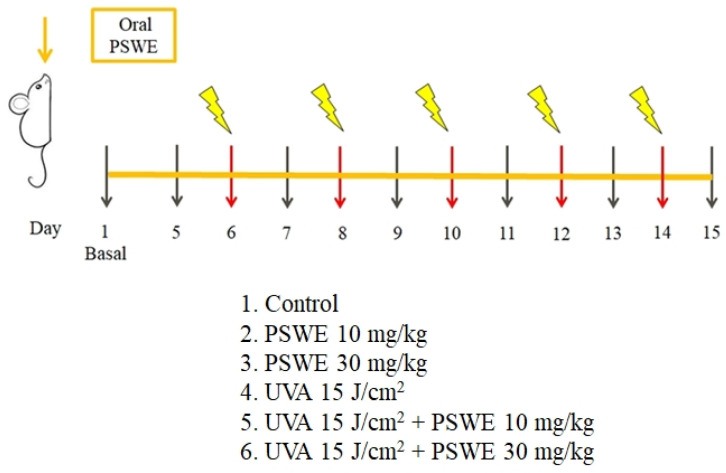
Study design of in vivo experimental model. The mice were divided into six groups, namely, 1. control group, 2. PSWE (10 mg/kg), 3. PSWE (30 mg/kg), 4. UVA 15 J/cm^2^ experimental group, 5. PSWE (10 mg/kg) + UVA 15 J/cm^2^ group, and 6. PSWE (30 mg/kg) + UVA 15 J/cm^2^ group. After the oral administration of PSWE at two different concentrations (10 mg/kg or 30 mg/kg), the skin physiological values of each mouse were measured on the 5th day, and then again on the 6th, 8th, 10th, 12th, and 14th days. Each mouse was irradiated with UVA 15 J/cm^2^ every day. PSWE was administered continuously for 15 days. The physiological values were measured on the second day of UVA irradiation, and the skin physiological conditions of the mice were recorded.

The animals used in this study were BALB/cAnN.Cg-Foxn1nu/CrlNarl hairless mice and were purchased from the National Laboratory Animal Center (NLAC). The breeding and handling of experimental animals follow the regulations of the Experimental Animal Center of Fu Jen Catholic University. Newly purchased mice were raised in a controlled environment, with 2–5 mice per cage, for a one-week adaptation period. The ambient temperature was set to 21 ± 2 °C, humidity of 50 ± 20%, lighting of 12 h light/12 h dark cycle per day, and ventilation frequency of 12 ± 2 times. Alfalfa-free food and drinking water ad libitum were provided. In this study, hairless mice aged 6–9 weeks were selected, and the mice were first divided into six groups, namely, 1. control group, 2. PSWE (10 mg/kg), 3. PSWE (30 mg/kg), 4. UVA 15 J/cm^2^ experimental group, 5. PSWE (10 mg/kg) + UVA 15 J/cm^2^ group, and 6. PSWE (30 mg/kg) + UVA 15 J/cm^2^ group. PSWE was dissolved in ddH_2_O and administered orally. The ultraviolet irradiation system used for animals is a Bio-Sun system illuminator (Vilber Lourmat, France). On the first day of the experiment, the skin physiologically relevant basal values of each mouse were measured, including transepidermal water loss (TEWL), melanin (melanin), red pigment (erythema), and skin hydration and blood flow (blood flow) as well as other parameters. Pictures were taken to record the changes in the appearance of the back skin. The temperature and humidity in the environment have a great impact on the parameters detected on the skin surface. Therefore, this study used a constant temperature and humidity chamber to maintain the temperature and humidity of the skin surface when evaluating the physiological parameters of the skin surface. Then, after the oral administration of PSWE at two different concentrations (10 mg/kg or 30 mg/kg), the skin physiological values of each mouse were measured on the 5th day, and then again on the 6th, 8th, 10th, 12th, and 14th days. Each mouse was irradiated with UVA 15 J/cm^2^ every day. PSWE was administered continuously for 15 days. The physiological values were measured on the second day of UVA irradiation, and the skin physiological conditions of the mice were recorded. Parameters and photographs were taken to observe the state of the dorsal skin of the mice. Finally, on the 15th day of the experiment, the mice were sacrificed under excessive anesthesia, and the dorsal skin tissue was removed for subsequent analysis, including H&E stain staining of sections and PCR detection of the promotion of inflammatory cells. Cytokine mRNA expression and protein phosphorylation were observed by Western blot (Figure 12).

### 4.7. Histopathological Analysis

The mouse dorsal skin was immersed in paraformaldehyde (PFA) diluted to a concentration of 4% in a buffer solution, placed at 4 °C for 8–12 h of tissue fixation step, and then, the tissue was taken out and directly embedded in paraffin. Paraffin blocks of the back skin were prepared by conventional methods and serially sliced at a thickness of 5 μm, followed by hematoxylin and eosin stain (H&E stain) for tissue section staining. Finally, the automatic fluorescent cell image observation system was used to obtain images of H&E staining of tissue sections.

### 4.8. Quantitative Polymerization Chain Reaction Technology (RT-qPCR)

The dorsal skin of mice was removed and immediately placed in a −80 °C refrigerator to prevent protein degradation. The grinding beads of appropriate size and number were added to the grinding tubes, along with 1 mL of Buffer RR of total RNA isolation kit (GeneDireX^®^, Vegas, NV, USA) and 10 μL of β-mercaptoethanol and 30 mg of the tissue sample, and then a grinder was used to grind this mixture, once every 15 s, and it was put in ice for 5 min as a cycle, a total of 6 times. Then, the tissue fluid was taken out, heated (70 °C, 30 min), and centrifuged using a low-temperature high-speed centrifuge (16,000 rcf, 10 min, 4 °C), and then centrifuged according to kit (GeneDireX^®^, Vegas, NV, USA) operating steps. A 500 μL volume of supernatant was added to the same proportion of 500 μL of 75% alcohol and shaken vigorously. A 500 μL volume of the sample mixture was drawn and put into the DR column, and then centrifuged at 14,000 rcf for 1 min. The waste liquid was removed after centrifugation. Buffer W1 was then added and centrifuged at 14,000 rcf for 1 min. Again, the waste liquid was removed, and Buffer W2 (diluted with alcohol) was added and centrifuged at 14,000 rcf to remove residual Buffer W2. Finally, an appropriate amount of Buffer RE was added to the DR column and allowed to stand for 2 min, then centrifuged at 14,000 rcf for 2 min. The procedure was repeated twice. Using this, we obtained the total intracellular RNA. The next step is to carry out the reverse transcription step. According to the operation process of the purchased iScriptTM cDNA Synthesis Kit (BIO-RAD, Hercules, CA, USA), reagents were added to convert RNA into cDNA [43]. Then, forward primer (forward primer) and reverse primer (reverse primer), 0.25 μL each (Table 1), ddH_2_O 7.5 μL, sample 2 μL, and finally fluorescence quantitative reagent (SYBR green) 10 μL were added. The prepared samples were placed into the instrument ABI StepOnePlusTM Real-time PCR System for quantitative PCR, which takes about 2 h, and can be analyzed after completion. In addition, the effect of PSWE on the expression of cytokine mRNA in UVA-irradiated HaCaT cells was also performed using the method described above. HaCaT cells were pretreated with different concentrations of PSWE (1, 10, and 30 μg/mL) for 1 h, and then, the cells were treated with UVA (15 J/cm^2^) for 24 h. Similar methods are described above and in depth in previously published papers [17,44].

### 4.9. Western Blot Analysis Test

Western blots were used to analyze the changes in various proteins in mouse tissues. The dorsal skin of the mouse was removed and divided into tubes, which were immediately placed in a −80 °C refrigerator to prevent protein degradation. The grinding beads, lysis buffer, and tissue samples were added to tubes, and then, a grinder was used to grind them for 15 s, and the ground mixture was put in ice for 5 min as a cycle, a total of 6 times. After grinding, a low-temperature high-speed centrifuge (13,200 rpm, 10 min, 4 °C) was used for centrifugation. After the centrifugation, the supernatant was extracted and placed on ice. Six new collection tubes with bovine serum albumin (BSA) + ddH_2_O were prepared and serially diluted 2, 1, 0.5, 0.25, 0.125, and 0.0625 times. A 200 µL volume from the Pierce BCA protein assay kit (Pierce, Rockford, IL, USA) was added in a ratio of 50:1 (A:B) to each tube and reacted in an incubator at 37 °C for 30 min. The enzyme-linked immunosorbent analyzer (ELISA reader) is used for analysis, and finally, the protein is quantified according to the required concentration of different proteins. The quantified sample and the sample buffer of the same proportion was added to the final collection tube. Then, the volumes required for the quantification of various proteins were sequentially loaded in 10% SDS–polyacrylamide gel, and the markers were used for electrophoresis, which took about 5 h. Afterward, the soaked cotton, the PVDF membrane (soaked in methanol for 10 min first), and the SDS–polyacrylamide gel were put together, and an appropriate amount of transfer buffer was added. The blotted membrane was cut to the required size, put in TBS-T (Tris-buffered saline/0.05%, tween20) solution containing 5% skimmed milk powder, and shaken for 1 h to avoid non-specific bonding. It was then washed with TBS-T 3 times, 10 min each time. The primary antibodies (primary antibodies) were added to 4 °C, placed in a cold room until the next day, and then washed with TBS-T 3 times, 10 min each time. After adding secondary antibodies (secondary antibodies) for 1 h, they were washed with TBS-T 3 times, 10 min each time. Finally, chromogenic reagents (agents A and B) were added. The membrane was placed in a chemical luminescence extraction system (BIOSTEP Celvin^®^) for shooting, and the expression intensity of the detected protein was quantified and analyzed. In the in vitro study, HaCaT cells were pretreated with different concentrations of PSWE (1, 10, and 30 μg/mL) for 1 h, and then, the cells were irradiated with UVA (15 J/cm^2^). Western blotting was also used to analyze changes in various proteins in cells. Relevant methods were similar to those described above and are described in depth in previously published papers [17,45].

### 4.10. Statistical Analysis

All the data were expressed by the Sigma-plot software 14.0. and the mean ± standard error (SE) was used as the representative value. The unpaired two-tailed test student *t*-test and two-way ANOVA were used for statistics. When a significant difference of *p* < 0.05 was found, it was marked with * and #. If the *p*-value was less than 0.01, it is marked with ** and ##.

## 5. Conclusions

Our study is the first to find that pear extract can effectively inhibit oxidative stress and damage caused by UVA. It is a natural extract with antioxidant properties and anti-aging activity that protects cells and strengthens the skin barrier. Therefore, it is deduced that further research on pear seed extract may be used to prevent skin-related diseases.

## Figures and Tables

**Table 1:** Mouse primer sequences for RT-PCR.

Genes	Primers	Sequence (5′-3′)
Mouse GAPDH	Forward Reverse	ACCCAGAAGACTGTGGATGG CACATTGGGGGTAGGAACAC
Mouse IL-1^®^	Forward Reverse	TGGACCTTCCAGGATGAGGACA GTTCATCTCGGAGCCTGTAGTG
Mouse IL-6	Forward Reverse	AGTTGCCTTCTTGGGACTGA TCCACGATTTCCCAGAGAAC
Mouse TNF-α	Forward Reverse	GGTGCCTATGTCTCAGCCTCTTTT GCCATAGAACTGATGAGAGGGAG

## Data Availability

Data are contained within the article.
